# Optimization of odour-baited resting boxes for sampling malaria vector, *Anopheles arabiensis *Patton, in arid and highland areas of Africa

**DOI:** 10.1186/1756-3305-3-75

**Published:** 2010-08-19

**Authors:** Eliningaya J Kweka, ,Beda J Mwang'onde, Aneth M Mahande

**Affiliations:** 1 Tropical Pesticides Research Institute, Division of Livestock and Human Diseases Vectors Control, Mosquito Section, P.O.Box 3024, Arusha, Tanzania; 2 Tropical Pesticides Research Institute, Mabogini Field Station, Moshi, Tanzania

## Abstract

**Background:**

Odour baited resting boxes are simple, reliable and important tools for sampling malaria vector mosquitoes in surveillance and control programmes in different parts of Africa. To optimize the use of cow urine baited resting boxes for sampling *An. arabiensis*, a community-based study was conducted in Mabogini hamlet in the Lower Moshi irrigation scheme area.

**Method:**

Experimental designs using 3 by 3 Latin square were conducted for twenty days to evaluate the following: i) the effect of different parameters in the sampling of mosquitoes using odour baited resting boxes; ii) the performance of odour baited traps under indoor and outdoor conditions and the effect of people sleeping indoors on mosquito density; iii) the effect of position in the placement of traps on collection of mosquitoes; and, iv) the efficiency of the trap outdoors at three different distances from the house wall. One extra house served as the sentinel house to monitor species abundance using a CDC-miniature light trap.

**Results:**

8581 mosquitoes were sampled by odour baited resting boxes of which, 8051 (93.82%) were *An. arabiensis *and 530 (6.18%) *Cx. quinquefasciatus*. The light trap collected 12,420 mosquitoes, of which 9442 (76.02%) were *An. arabiensis*, 126 (1.01%) *An. funestus *group, 230 (1.85%) *An. rufipes *and 2622 (21.11%) *Cx. quinquefasciatus*. The best height for outdoor mosquitoes sampling was 15 cm and 220 cm while indoors was 105 cm. The difference in mosquito collection between different outdoor and indoor heights was statistically significant (p < 0.0001). The optimal outdoor location of odour baited resting boxes from the wall of the house was 3 m.

**Conclusions:**

The results of these studies demonstrate an optimal method for sampling during surveillance and control programmes in rural villages of highlands and arid areas of Africa using inexpensive baits and boxes.

## Background

Hematophagus insects locate their hosts following odours emanated by the hosts [1-5]. The *Anopheles gambiae *complex comprises two major malaria vector species *An. gambiae *Giles and *An. arabiensis *Patton while the *An. funestus *group include the major malaria vector, *An. funestus *Giles [6]. All three species are abundant in sub-Saharan Africa [6]. *An. gambiae *ss are anthropophilic while *An. arabiensis *are both anthropophilic as well as zoophilic depending on geographical location [6,7]. Human sweats and other animal body products attract haematophagus insects, such as tsetse flies[8,9]; mosquitoes [10-13] and sand flies [14]. Knowledge of these attractants plays a vital role in reducing human vector contact and in conducting surveillance studies. Resting boxes, either baited or un-baited, have been demonstrated to be suitable for the sampling of malaria vectors in different parts of Africa [15,16]. Fresh or decayed cattle urine baited resting boxes are effective in sampling *An. Arabiensis*, with efficacy even after seven days post-treatment [15,17].

This study deployed the fresh cattle urine in wet black cotton cloth to study the optimal trap placement and validate the effectiveness of the methodology. Three sets of experiments were conducted to determine: i) if traps with urine as bait perform better indoors or outdoors, and if people sleeping indoors had effects on the mosquito density; ii) the best height of trapping mosquitoes indoors and outdoors; and iii) the efficiency of the trap located in three different distances from the wall of the house.

## Materials and methods

### Study Area Description

**Figure 1:**
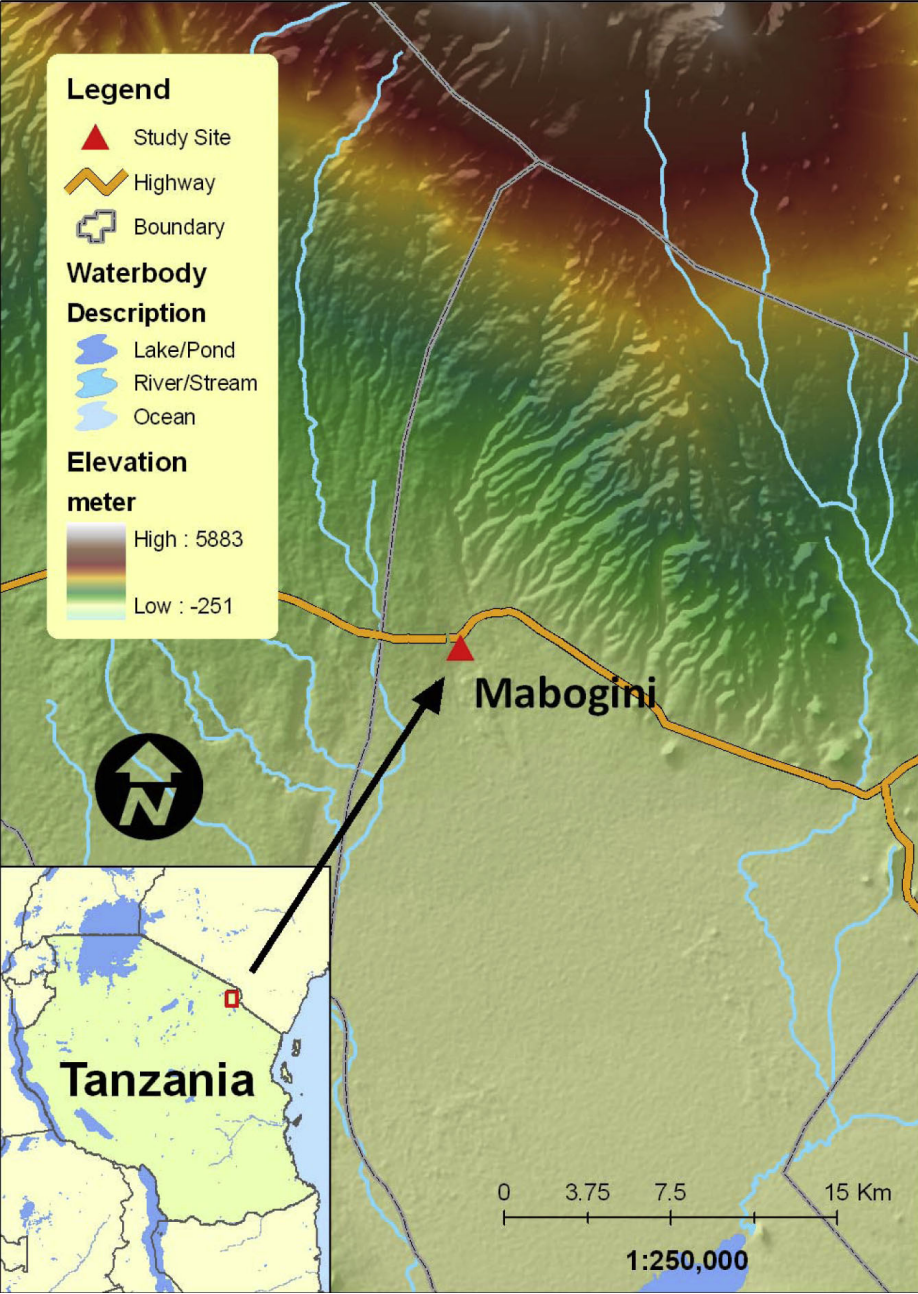
**Map showing the area where the sampling was performed at Point D in Lower Moshi irrigation schemes in Northern Tanzania**.

The study was conducted during the paddy transplantation season in the lower Moshi irrigation schemes, located 15 Km southern part of Moshi Town at the foot of the slopes of Mount Kilimanjaro, Northern Tanzania (3°21'S, 37°21'E). The study area is shown in Figure 1.

### House selection and characterization

Ten houses were surveyed during the preparation stage of the study using CDC-miniature light traps for collecting mosquitoes from the surveyed houses. Four houses with high mosquito density were selected for the main study. In these selected houses, parameters such as eave sizes, number of windows, wall and roof types and number of people sleeping in the house during experimental nights were recorded and compared with indoor mosquito density.

### Experimental design

The study was a cross-sectional design in 3 by 3-Latin square for 20 days with an extra house fitted with CDC light trap for mosquitoes species abundance monitoring. One replicate was conducted for a period of three days; the study had a total of 6 replicates for 18 days. Two extra days were added to complement the lost time in two experimental days. In the first experiment, two baited resting boxes were used indoors and outdoors and placed at three different heights: 15 cm (H1); 105 cm (H2); and 220 cm (H3). Each house was used with one pair of boxes per night at one height.

The second experiment was designed to determine the optimal outdoor location for odour baited resting boxes by selecting three distances from the external wall of the house: 0.3 m; 3 m; and 5 m. Each distance was tested per house per night in 3 by 3 Latin square for 20 days. The experiments were conducted for a period of twenty days between January and February, 2010.

### Animal husbandry, urine collection and preparation of the boxes

Urine was collected in the morning from a mature female zebu cow and stored in a refrigerator at 4°C until the start of the experiment on the same evening. Boxes were prepared as previously described [15,17].

### Mosquito sampling

Trapping of mosquitoes by odour baited resting boxes and CDC-miniature light trap started at 1800 hours. The trapped mosquitoes were collected at 0600 hours in the following morning using a mechanical aspirator from the odour baited resting boxes and transferred to labelled cups. The Identification key [7] was used to identify mosquitoes in the laboratory. The abdominal conditions were identified as per World Health Organization manual [18].

### Statistical Analysis

Data were encoded in Excel spreadsheet before exporting to PWAS Statistics 18.0 (SPSS Inc., Chicago, IL) for analysis. Due to the high variability in mosquito density in each sampling point, all data were log transformed (log (n+1)) before analysis to normalise the data. General linear model univariate analytical procedure was used to assess the effect of odour baited resting boxes at different heights, house position and days of mosquito sampling. Paired sample T-test was used to compare the density of mosquitoes collected indoors and outdoors in odour baited resting boxes. ANOVA test was used to compare the density differences of mosquitoes with the height of the traps from indoor and outdoor locations. ANOVA was also used to compare the variations of mosquitoes density collected from different distances from the experimental house walls (0.3 m, 3 m and 5 m) and paired t-test was used to compare the mean density between each distance. The effect of the evaluated sampling tool was considered to be significant at p < 0.05.

### Ethical considerations

The Ethics Committee of the Tropical Pesticides Research Institute approved the study. The objectives of the study were discussed with residents of the hamlet and a signed written consent was obtained from the head of each participating household.

## Results

### Mosquito species abundance, house characteristics and factors associated with variation in mosquito density

The CDC-miniature light trap sampled 12,420 mosquitoes of which 9,442 (76.02%) were *An. gambiae s.l*, 126 (1.01%) *An. funestus *group, 230 (1.85%) *An. rufipes *and 2,622 (21.11%) *Cx.quinquefasciatus *(Additional file 1: Table S1). Odour baited resting boxes collected 8581 mosquitoes of which, 8,051 (93.82%) were *An. gambiae s.l *and 530 (6.18%) were *Cx. quinquefasciatus *(Additional file 2: Table S2). All *An. gambiae *s.l collected were considered as *An. arabiensis *according to previous molecular identification done by Ijumba, *et al *[19] in the same study area. The abdominal conditions of all *An. arabiensis *from the CDC light trap were unfed while those from odour baited resting boxes had different abdominal conditions (Table 1). The experimental houses all represented the typical characteristics of the traditional houses found in lower Moshi irrigation scheme (Table 2).

**Table 1:** Abdominal conditions of female *An. arabiensis *and *Cx. quinquefasciatus *sampled by light trap and odour baited resting boxes in selected houses.

Method	Species	Unfed	Fed	Semi gravid	Gravid
CDC light trap	*An. arabiensis*	9442 (44.97%)	0	0	0
	
	*An. funestus *group	126 (0.60%)	0	0	0
	
	*An.rufipes*	320 (1.52%)	0	0	0
	
	*Cx. quinquefasciatus*	2532 (12.06%)	0	0	0

Odour baited resting boxes	*An. arabiensis*	820 (3.90%)	3119 (14.89%)	2984 (14.21%)	1128 (5.37%)
	
	*Cx. quinquefasciatus*	180 (0.86%)	94 (0.45%)	220 (1.05%)	36 (0.17%)

The percentage is derived from overall sampled mosquitoes by both methods

**Table 2:** Characteristics of houses used in the evaluation of odour baited resting boxes.

House ID	Number of occupants/night	Light and Fuel source	Roof type	Wall type	Eave size (cm)	Number of windows per room	Cooking place
HS1	3	Kerosene and charcoal	Iron sheets	Mud wall	15	1	outdoors

HS2	4	Kerosene and firewood	Iron sheets	Burnt bricks	13	2	outdoors

HS3	6	Kerosene and firewood	Iron sheets	Burnt bricks	10	1	outdoors

HS4	4	Kerosene and charcoal	Iron sheets	Cement bricks	21	1	outdoors

The mosquito density collected by CDC light trap in one house and resting boxes in three houses varied significantly, mainly due to the vertical (height) positioning of the trap in both indoor and outdoor (DF = 3, F = 26.034, p < 0.0001). The number of days had no effect for both indoor (DF = 19, F = 0.959, P = 0.694) and outdoor (DF = 19, F = 1.771, P = 0.113) positions. The location of house also had no effect on density of mosquitoes indoor (DF = 3, F = 0.866, p = 0.464) and outdoor (DF = 3, F = 1.198, P = 0.319).

### Performance of the trap between outdoor, indoor, and effect of human inhabitants sleeping indoors

The indoor and outdoor mean densities (95% confidence intervals in parenthesis) were found to be 0.81(0.64-0.98) and 2.14(1.97-2.41), respectively. The densities of *An. gambiae *s.l mosquitoes sampled indoor and outdoor using odour baited resting boxes were statistically different (t = 4.477, DF = 59, P < 0.0001). The overall indoor mosquito density was higher than outdoor.

### Optimal height of placement of resting boxes in indoor and outdoor conditions

**Figure 2:**
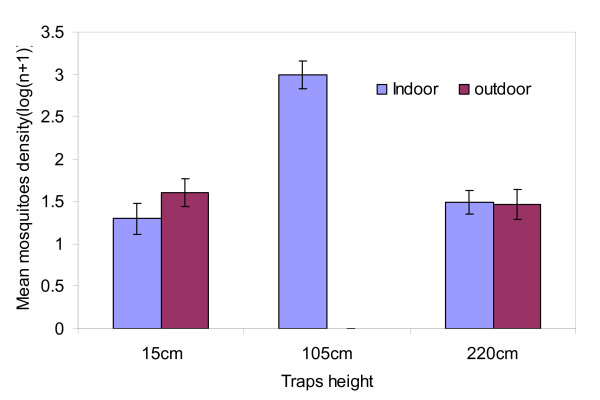
**The mean number of mosquitoes collected at different heights for both indoor and outdoor using the odour baited resting boxes**.

The height of the odour baited resting boxes had an impact on the number of collected mosquitoes in both indoor (DF = 2, F = 143.607, P < 0.0001) and outdoor (DF = 2, F = 180.986, P < 0.0001) locations. Indoors, H2 (105 cm) positioned odour baited resting boxes collected more mosquitoes than H1 (15 cm) and H3 (220 cm). Outdoors, H1 sampled more mosquitoes than H3 while H2 sampled no mosquitoes (Figure 2). In using paired t-test for sampled means, the difference between indoor resting boxes, H1 and H2 was significant (p < 0.0001). The same was true for H2 and H3 (p < 0.0001) and for H1 and H3 too (P = 0.05). In the outdoor positions, the difference between H1 and H2 (P < 0.0001) and for H2 and H3 (p < 0.0001) was significant. However, H1 and H3 showed no significant differences (P = 0.08).

### Efficiency of the trap in collecting outdoor mosquitoes at different distances from the house

**Figure 3:**
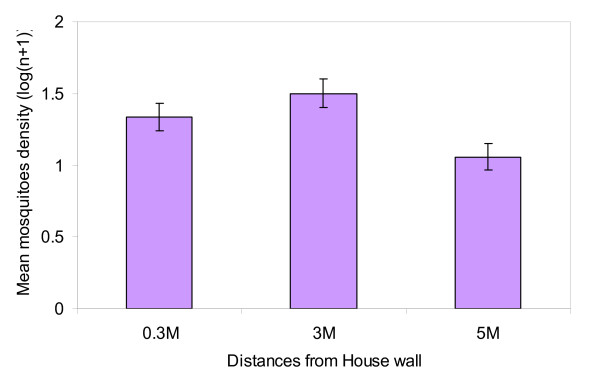
**The mean variation of mosquitoes sampled outdoors at different distances from the house wall**.

The number of mosquitoes collected from the baited resting boxes placed at 0.3 m, 3 m and 5 m from the wall of the house were statistically significant (DF = 2, F = 17.3592, P < 0.0001). In comparing densities between distances with t-test paired two samples of means, the density difference between 0.3 m and 3 m was significantly different (df = 59, t = 1.67, p < 0.0001) while 0.3 m and 5 m was not significant (df = 5, t = 1.67, p = 0.09). The number of mosquitoes collected at 3 m and 5 m were significantly different (df = 5, t = 1.67, p < 0.0001). The highest density of mosquitoes was found in odour baited resting boxes placed at 3 m away from the wall of each house (Figure 3 and Additional file 3: Table S3).

## Discussion

This study has shown that a resting box baited with cattle urine odour is efficient in collecting female *An. arabiensis *and *Cx. quinquefasciatus*. This efficiency is dependent upon the height at which the resting boxes are placed for indoors and outdoors and the distance from the wall of the house. The addition of cattle urine in resting boxes showed significant improvement in the efficiency of the traps in collecting mosquitoes. These observations are similar to the observations obtained from previous studies [15,17]. The number of people sleeping in the house, house positions and days of collections had no effect. The presence of odour baited resting boxes did not represent a nuisance to people sleeping on their beds. The outside baited resting boxes had more mosquitoes (*An. gambiae *s.l) at the height of 15 cm and at 220 cm (eave position) than at 105 cm (window position, at intermediate height). The highest density for indoor sampling was at the height of 105 cm (window position) compared to 15 cm and 220 cm. This effect may result from incoming morning sunshine through the gaps in the window that may have caused most of the mosquitoes to fly out and trapped resting in traps. The boxes placed at 3 m away from the house wall had higher density than those placed at 0.3 m and 5 m (Figure 3). For optimal use, it is advised to place the odour baited resting boxes for trapping *An. arabiensis *along the window level during indoor sampling and 15 cm and adjacent to the eave position for outdoors. The 3 m distance from the house wall is recommended for placing odour baited resting boxes for efficient trapping of *An. arabiensis *seeking foraging places.

Further studies are needed to integrate baits, such as cattle urine, and entomopathogenic fungi or non-repellent insecticides in order to pave the way towards better control of malaria. Non-irritant insecticides and entomopathogenic fungi are effective against mosquitoes in both laboratory and field evaluation [20-24]. The treatment of the odour baited resting boxes with insecticides and fungus and its placement in optimal positions can play a major role in increasing mosquito mortality. Once these odour baited boxes are deployed, other factors for reducing entry of mosquitoes, such as house modifications, should be emphasized [25,26].

The CDC-miniature light traps are more efficient than the baited resting boxes in the density and diversity of species of mosquitoes collected. However, baited resting boxes are better in sampling fed and gravid mosquitoes, which are more important in addressing malaria transmission in terms of blood meal sources and parity rates (Table 1). The urine baited traps provide a useful alternative tool for sampling malaria vector mosquitoes. This method can also be useful to infect blood fed, semi and full gravid mosquitoes with entomopathogenic fungi [20,24]. The higher density of semi-gravid and gravid mosquitoes using this baiting method is useful in evaluating oviposition behavior of malaria vectors and in determining the best control method in targeting ovipositing mosquitoes.

This study demonstrated the optimal height and distance from the house wall for surveillance or the control of *An. arabiensis *that may find use in different highlands and arid areas of African where these vectors dominate. This methodology is also promising because it allows sampling of mosquito vectors without risking human volunteers to infectious mosquito bites. The boxes and cow urine are easily accessible locally and are abundant in rural communities where the method is potentially useful. The trap does not need skilled personnel to operate and are environmentally friendly. Because these traps collect live mosquitoes, they can be used in biological studies to raise colonies for studies on parity rates, insecticidal susceptibility and other scientific/research interests.

## Conclusion

This study demonstrated the usefulness of odour baited boxes for sampling mosquitoes and the optimum location for placement in indoor and outdoor positions. Optimal collection rates outdoors was at a distance of 3 m from house wall. The best height in outdoor placement was 15 cm and 220 cm while for indoor was 105 cm.

## Competing interests

The authors declare that they have no competing interests.

## Authors' contributions

EJK conceived and designed the experiments, analyzed and interpreted the data. BJM and MAM performed the experiments. EJK and BJM wrote the paper. All authors approved the manuscript for submission.

## Supplementary Material

Additional file 1 Supplementary Table S1: Total female mosquitoes collected by CDC light trap for 20 days of monitoring the mosquito densities in houses. Click here for file

Additional file 2 Supplementary Table S2: The total number of female mosquitoes collected by Odour Baited Resting Boxes (OBRB) at different heights for both indoor and outdoors for 20 days. Click here for file

Additional file 3 Supplement Data Table S3: The number of the female *An. arabiensis *sampled during 20 days of experiments by odour baited resting boxes placed outdoors at different distances from the wall of each house. Click here for file
