# Supplementary material for: Optimization of odour-baited resting boxes for sampling malaria vector, Anopheles arabiensis Patton, in arid and highland areas of Africa
Source: Parasit Vectors. 2010 Aug 19;3:75. doi: 10.1186/1756-3305-3-75 (PMC2933686; doi:10.1186/1756-3305-3-75)
Supplement: Additional file 1 — Supplementary Table S1: Total female mosquitoes collected by CDC light trap for 20 days of monitoring the mosquito densities in houses. [file 1756-3305-3-75-S1.DOC]

**Supplement data Table S1**:

| **Days** | ***An.gambiae s.l*** | ***An.funestus*** | ***An.rufipes*** | ***Cx. quinquefasciatus*** |
| --- | --- | --- | --- | --- |
| 1 | 490 | 9 | 25 | 169 |
| 2 | 588 | 4 | 12 | 198 |
| 3 | 479 | 3 | 13 | 108 |
| 4 | 549 | 8 | 18 | 184 |
| 5 | 492 | 13 | 25 | 63 |
| 6 | 671 | 8 | 15 | 59 |
| 7 | 487 | 5 | 30 | 81 |
| 8 | 390 | 3 | 8 | 144 |
| 9 | 561 | 6 | 9 | 93 |
| 10 | 598 | 7 | 12 | 208 |
| 11 | 453 | 9 | 11 | 97 |
| 12 | 336 | 8 | 14 | 189 |
| 13 | 354 | 9 | 28 | 196 |
| 14 | 529 | 8 | 29 | 126 |
| 15 | 295 | 9 | 12 | 134 |
| 16 | 387 | 7 | 11 | 43 |
| 17 | 299 | 3 | 9 | 94 |
| 18 | 682 | 2 | 7 | 86 |
| 19 | 408 | 1 | 3 | 91 |
| 20 | 394 | 4 | 29 | 169 |
| **Total females** | **9442** | **126** | **320** | **2532** |
