# Supplementary material for: Optimization of odour-baited resting boxes for sampling malaria vector, Anopheles arabiensis Patton, in arid and highland areas of Africa
Source: Parasit Vectors. 2010 Aug 19;3:75. doi: 10.1186/1756-3305-3-75 (PMC2933686; doi:10.1186/1756-3305-3-75)
Supplement: Additional file 2 — Supplementary Table S2: The total number of female mosquitoes collected by Odour Baited Resting Boxes (OBRB) at different heights for both indoor and outdoors for 20 days. [file 1756-3305-3-75-S2.DOC]

**Supplementary Table S2:**

| **DAYS** | **Indoor** | | | | | **Outdoor** | | | | | | |
| --- | --- | --- | --- | --- | --- | --- | --- | --- | --- | --- | --- | --- |
|  | **OBRB-15cm** | | **OBRB-105 cm** | | **OBRB-220cm** | | **OBRB-15cm** | | **OBRB-105 cm** | | **OBRB-220cm** | |
|  | *An.gambiae s.l* | *Cx.quinquefasciatus* | *An.gambiae s.l* | *Cx.quinquefasciatus* | *An.gambiae s.l* | *Cx.quinquefasciatus* | *An.gambiae s.l* | *Cx.quinquefasciatus* | *An.gambiae s.l* | *Cx.quinquefasciatus* | *An.gambiae s.l* | *Cx.quinquefasciatus* |
| 1 | 63 | 5 | 115 | 6 | 106 | 5 | 133 | 9 | 0 | 0 | 102 | 8 |
| 2 | 41 | 2 | 94 | 7 | 49 | 4 | 102 | 4 | 0 | 0 | 98 | 4 |
| 3 | 29 | 5 | 86 | 5 | 43 | 6 | 76 | 6 | 0 | 0 | 69 | 3 |
| 4 | 74 | 4 | 159 | 3 | 61 | 5 | 73 | 8 | 0 | 0 | 88 | 7 |
| 5 | 62 | 6 | 78 | 8 | 59 | 7 | 87 | 6 | 0 | 0 | 130 | 8 |
| 6 | 59 | 2 | 63 | 8 | 63 | 8 | 73 | 11 | 0 | 0 | 112 | 6 |
| 7 | 40 | 3 | 71 | 10 | 41 | 9 | 104 | 8 | 0 | 0 | 151 | 3 |
| 8 | 39 | 1 | 102 | 5 | 39 | 12 | 99 | 9 | 0 | 0 | 181 | 9 |
| 9 | 38 | 0 | 116 | 6 | 81 | 1 | 108 | 11 | 0 | 0 | 111 | 4 |
| 10 | 40 | 0 | 103 | 9 | 42 | 2 | 127 | 24 | 0 | 0 | 91 | 6 |
| 11 | 45 | 5 | 83 | 0 | 49 | 0 | 131 | 0 | 0 | 0 | 108 | 2 |
| 12 | 48 | 9 | 57 | 4 | 41 | 0 | 98 | 9 | 0 | 0 | 59 | 3 |
| 13 | 43 | 11 | 127 | 0 | 51 | 0 | 87 | 0 | 0 | 0 | 93 | 0 |
| 14 | 46 | 0 | 152 | 0 | 43 | 0 | 79 | 0 | 0 | 0 | 90 | 0 |
| 15 | 61 | 4 | 100 | 4 | 54 | 6 | 125 | 15 | 0 | 0 | 84 | 3 |
| 16 | 65 | 3 | 86 | 5 | 50 | 4 | 138 | 18 | 0 | 0 | 97 | 3 |
| 17 | 51 | 2 | 94 | 6 | 39 | 6 | 145 | 22 | 0 | 0 | 56 | 2 |
| 18 | 49 | 1 | 104 | 7 | 30 | 3 | 151 | 4 | 0 | 0 | 60 | 6 |
| 19 | 57 | 4 | 112 | 9 | 28 | 3 | 107 | 8 | 0 | 0 | 49 | 1 |
| 20 | 30 | 3 | 141 | 8 | 33 | 9 | 113 | 8 | 0 | 0 | 41 | 2 |
| **Total females** | **980** | **70** | **2043** | **110** | **1002** | **90** | **2156** | **180** | **0** | **0** | **1870** | **80** |
