# Supplementary material for: Optimization of odour-baited resting boxes for sampling malaria vector, Anopheles arabiensis Patton, in arid and highland areas of Africa
Source: Parasit Vectors. 2010 Aug 19;3:75. doi: 10.1186/1756-3305-3-75 (PMC2933686; doi:10.1186/1756-3305-3-75)
Supplement: Additional file 3 — Supplement Data Table S3: The number of the female An. arabiensis sampled during 20 days of experiments by odour baited resting boxes placed outdoors at different distances from the wall of each house. [file 1756-3305-3-75-S3.DOC]

| **Days** | **Distance 1(0.3M)** | **Distance 2(3M)** | **Distance(5M)** |
| --- | --- | --- | --- |
| 1 | 55 | 82 | 27 |
| 2 | 30 | 45 | 15 |
| 3 | 13 | 19 | 6 |
| 4 | 53 | 79 | 26 |
| 5 | 13 | 20 | 7 |
| 6 | 30 | 44 | 15 |
| 7 | 33 | 50 | 17 |
| 8 | 66 | 98 | 33 |
| 9 | 20 | 30 | 10 |
| 10 | 21 | 32 | 11 |
| 11 | 24 | 35 | 12 |
| 12 | 32 | 49 | 16 |
| 13 | 13 | 19 | 6 |
| 14 | 22 | 33 | 11 |
| 15 | 30 | 45 | 15 |
| 16 | 37 | 55 | 18 |
| 17 | 17 | 25 | 8 |
| 18 | 19 | 29 | 10 |
| 19 | 16 | 24 | 8 |
| 20 | 46 | 69 | 23 |
| **Totals** | **588** | **882** | **294** |

**Supplement Data Table S3**:
